# A Nogo-Like Signaling Perspective from Birth to Adulthood and in Old Age: Brain Expression Patterns of Ligands, Receptors and Modulators

**DOI:** 10.3389/fnmol.2018.00042

**Published:** 2018-02-19

**Authors:** Gabriella Smedfors, Lars Olson, Tobias E. Karlsson

**Affiliations:** Department of Neuroscience, Karolinska Institute, Stockholm, Sweden

**Keywords:** development, Nogo-A, NgR1, Lingo-1, Troy, OMgp, Olfactomedin, hippocampus

## Abstract

An appropriate strength of Nogo-like signaling is important to maintain synaptic homeostasis in the CNS. Disturbances have been associated with schizophrenia, MS and other diseases. Blocking Nogo-like signaling may improve recovery after spinal cord injury, stroke and traumatic brain injury. To understand the interacting roles of an increasing number of ligands, receptors and modulators engaged in Nogo-like signaling, the transcriptional activity of these genes in the same brain areas from birth to old age in the normal brain is needed. Thus, we have quantitatively mapped the innate expression of 11 important genes engaged in Nogo-like signaling. Using *in situ* hybridization, we located and measured the amount of mRNA encoding Nogo-A, OMgp, NgR1, NgR2, NgR3, Lingo-1, Troy, Olfactomedin, LgI1, ADAM22, and MAG, in 18 different brain areas at six different ages (P0, 1, 2, 4, 14, and 104 weeks). We show gene- and area-specific activities and how the genes undergo dynamic regulation during postnatal development and become stable during adulthood. Hippocampal areas underwent the largest changes over time. We only found differences between individual cortical areas in Troy and MAG. Subcortical areas presented the largest inter-regional differences; lateral and basolateral amygdala had markedly higher expression than other subcortical areas. The widespread differences and unique expression patterns of the different genes involved in Nogo-like signaling suggest that the functional complexes could look vastly different in different areas.

## Introduction

**Figure 1:**
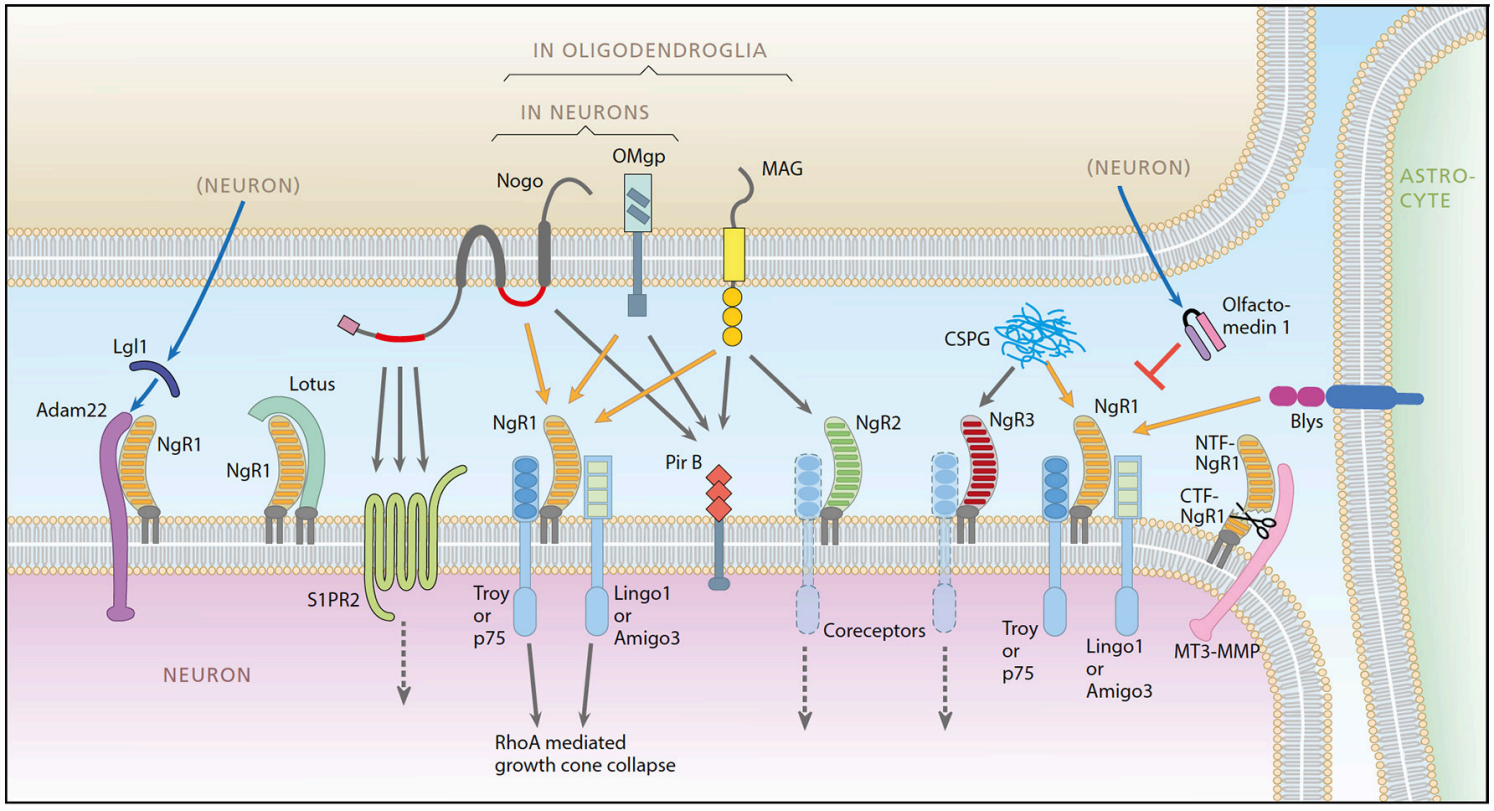
A schematic representation of the Nogo system. Ligands: Nogo-A, OMgp, MAG, Blys. Receptors: NgR1, NgR2, NgR3, PirB, ADAM22, S1PR2. Coreceptors: Troy, P75, Lingo-1, Amigo3. Modulators: LOTUS, LgI1, Olfactomedin, CSPG, MT3-MMP. Picture from Karlsson et al. (2017).

The nerve-growth inhibitory ligand Nogo-A was first identified in CNS myelin in 1988 (Caroni and Schwab, 1988a,b) and later shown to be expressed also in neurons (Chen et al., 2000; Josephson et al., 2001). Currently, known ligands, receptors, co-receptors, and modulators participating in Nogo-like signaling encompass some 20 proteins (Mironova and Giger, 2013; Schwab and Strittmatter, 2014; Sui et al., 2015; Seiler et al., 2016). Together, they regulate development, adult structural synaptic plasticity and memory (McGee et al., 2005; Mingorance-Le Meur et al., 2007; Giger et al., 2010; Akbik et al., 2013; Mironova and Giger, 2013; Nordgren et al., 2013; Schwab and Strittmatter, 2014; Karlsson et al., 2016, 2017; Zagrebelsky et al., 2017), including appropriate motor learning (Zemmar et al., 2014). The system also affects recovery from injury, such as axonal regeneration (Chen et al., 2017), and is involved in CNS reactions to injury and disease, including multiple sclerosis, schizophrenia, intracerebral tumors and stroke (Eslamboli et al., 2006; Cheatwood et al., 2008; Jung et al., 2011; Xiong et al., 2012; Willi and Schwab, 2013). Nogo-A can bind to Nogo receptor 1 (NgR1) through an extracellular loop, activating the NgR1 signaling complex (including coreceptors) resulting in nerve growth inhibition via the RhoA pathway, visualized in Figure 1 (Fournier et al., 2001; Niederöst et al., 2002; Sui et al., 2015). The signaling system has grown more complex as exemplified by the fact that Nogo-A can also bind to paired immunoglobulin-like receptor B (PirB), sphingosine-1-phosphate receptor 2 (S1PR2), and Syndecans (GrandPré et al., 2002; Atwal et al., 2008; Kempf et al., 2014, 2017). Furthermore, NgR1 can also be activated by myelin associated glycoprotein (MAG) (Domeniconi et al., 2002), oligodendrocyte myelin glycoprotein (OMgp) (Wang et al., 2002), chondroitin sulfate proteoglycans (CSPGs, extracellular matrix proteins known to inhibit neuronal growth) (Dickendesher et al., 2012) and the immune system component B lymphocyte stimulator (BLYS) (Zhang et al., 2009). Nogo-A is the most potent inhibitory agent of these ligands (Cafferty et al., 2010).

NgR1 is a glycophoshpatidylinositol (GPI)-linked receptor, and needs coreceptors to transduce signals. Two alternative coreceptor complexes have been identified: P75 or Troy together with Lingo-1 or Amigo3 (Wang et al., 2002; Mi et al., 2004; Park et al., 2005; Saha et al., 2014). Lingo-1 is specific to CNS neurons (Mi et al., 2004) and widely expressed in the CNS (Okafuji and Tanaka, 2005; Barrette et al., 2007). Since Lingo-1 mRNA is more broadly expressed than NgR1 and P75 (Lee et al., 2007; Llorens et al., 2008) its actions appear to extend beyond those mediated by the inhibitory NgR1-coreceptor complex. The Troy coreceptor is also widely expressed in the CNS, both in the embryo and the adult (6–12 weeks old) (Park et al., 2005; Hisaoka et al., 2006), while p75, which also serves as the low affinity NGF receptor, has a very limited CNS expression (Boskovic et al., 2014). Amigo 3 has a very weak expression in the brain^1^.

NgR2 and NgR3 are two homologous variants of NgR1 (Laurén et al., 2003). NgR2 can bind MAG (Venkatesh et al., 2005), NgR3 serves as a receptor for CSPGs (Dickendesher et al., 2012). It should be noted that NgR3 is not specific to the CNS, as it can be found in skeletal muscle and the heart (Huber et al., 2002). NgR1 antagonists are associated with growth cone stability and include Olfactomedin-1 (subsequently referred to as Olfactomedin in this article), lateral olfactory tract usher substance (LOTUS) and Leucine-rich glioma inactivated 1 (LGI1) which in turn is facilitated by ADAM22 (Thomas et al., 2010; Sato et al., 2011; Nakaya et al., 2012).

So far, the Nogo-system has been investigated mostly with a focus on individual or smaller groups of components (Habib et al., 1998; Huber et al., 2002; Josephson et al., 2002; Tozaki et al., 2002; Richard et al., 2005; Barrette et al., 2007; Haybaeck et al., 2012; Kumari and Thakur, 2014). The expression has also been investigated in response to activation and/or injury (Josephson et al., 2003; Mingorance et al., 2004; Cafferty et al., 2010; Borrie et al., 2012). Our lab recently described how a larger group of genes in the Nogo-system are affected during the first 3 days after strong neuroexcitation by kainic acid in adult mice, as a basis for hypotheses about the summed effects on neural plasticity of multiple gene alterations (Karlsson et al., 2017). To understand how the Nogo-like signaling genes may interact during brain development, adulthood and aging, we have now investigated the spatial and temporal patterns of transcriptional activity of 11 genes involved in Nogo-like signaling at six time-points in life, in 18 brain regions. We find that there is a marked alteration of expression levels in different areas during development. In adulthood and aging the system is remarkably stable, but ready to rapidly change in response to neuronal activity (Josephson et al., 2003; Karlsson et al., 2017).

## Methods

### Ethics statement

The experiments were approved by the Stockholm North animal ethics committee (N246/15).

### Animals

Male and female naïve C57BL/6 mice (Charles River) were housed in ventilated boxes with at least one and a maximum of four cohabitants. Food and water was administered *ad libitum*. The cages were enriched with a paper house and tissue paper for nesting. Cages were kept in rooms with a 12/12 h light/dark cycle at 22–23°C and a relative humidity of 60%. The first month after birth is a critical period for brain development and maturation during which the brain is known to undergo substantial changes. Thus, we studied four stages from the first month of life: newborn, 1, 2, and 4 weeks old animals. From the second month onwards, known changes of brain development are minor. We chose to study 14 week old mice, representing “mature adults.” We were also interested in aging, and chose to study very old animals to optimize chances of finding age-related changes.

### *In situ* hybridization

**Figure 2:**
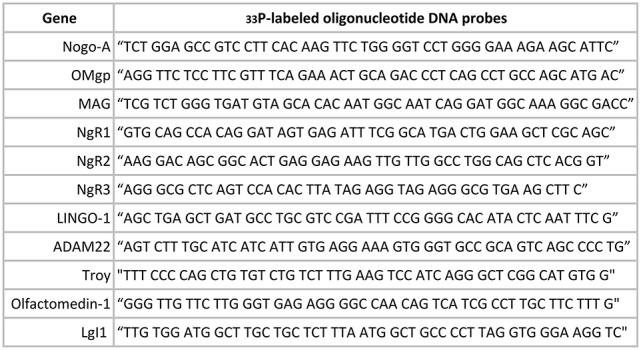
Oligonucleotide DNA probes.

In order to perform *in situ* hybridization (ISH), tissue was collected after sacrifice through decapitation at six time points during postnatal development and in adult and aged animals (P0, 1, 2, 4 14 weeks, and 2 years (104 w); *n* = 3–12 per time point). Tissue was fresh frozen and serial coronal 14 μm sections were collected with a cryostat (Microm HM500M; Microm HM560; Thermo Scientific). The sections were instantly mounted on coated slides (VWR Superfrost Plus Micro Slide). For each gene every 10th section was used for the analysis. High stringency ISH was performed based on a protocol from Dagerlind et al. (1992) with oligonucleotide probes (see Figure 2). After ^33^P labeling, and prior to being used for hybridization, each probe was measured for radioactive strength. Air-dried, hybridized sections were exposed to film for autoradiograhy (Carestream Kodak BioMax MR-Film, Sigma-Aldrich) after exposure time had been established with the help of phosphoimaging (Fujix BAS-3000; Fuji Photo Film Co, Tokyo, Japan), to determine quality and strength of isotope labeling. Film exposure times were 8–57 days based on gene and probe strength. Films were developed and scanned (Epson Perfection V750 Pro, Dual lens system, High pass optics; Digital ICE Technologies, Long Beach, CA, USA). The following DNA probes are listed in Figure 2.

### Quantification of mRNA

**Figure 3:**
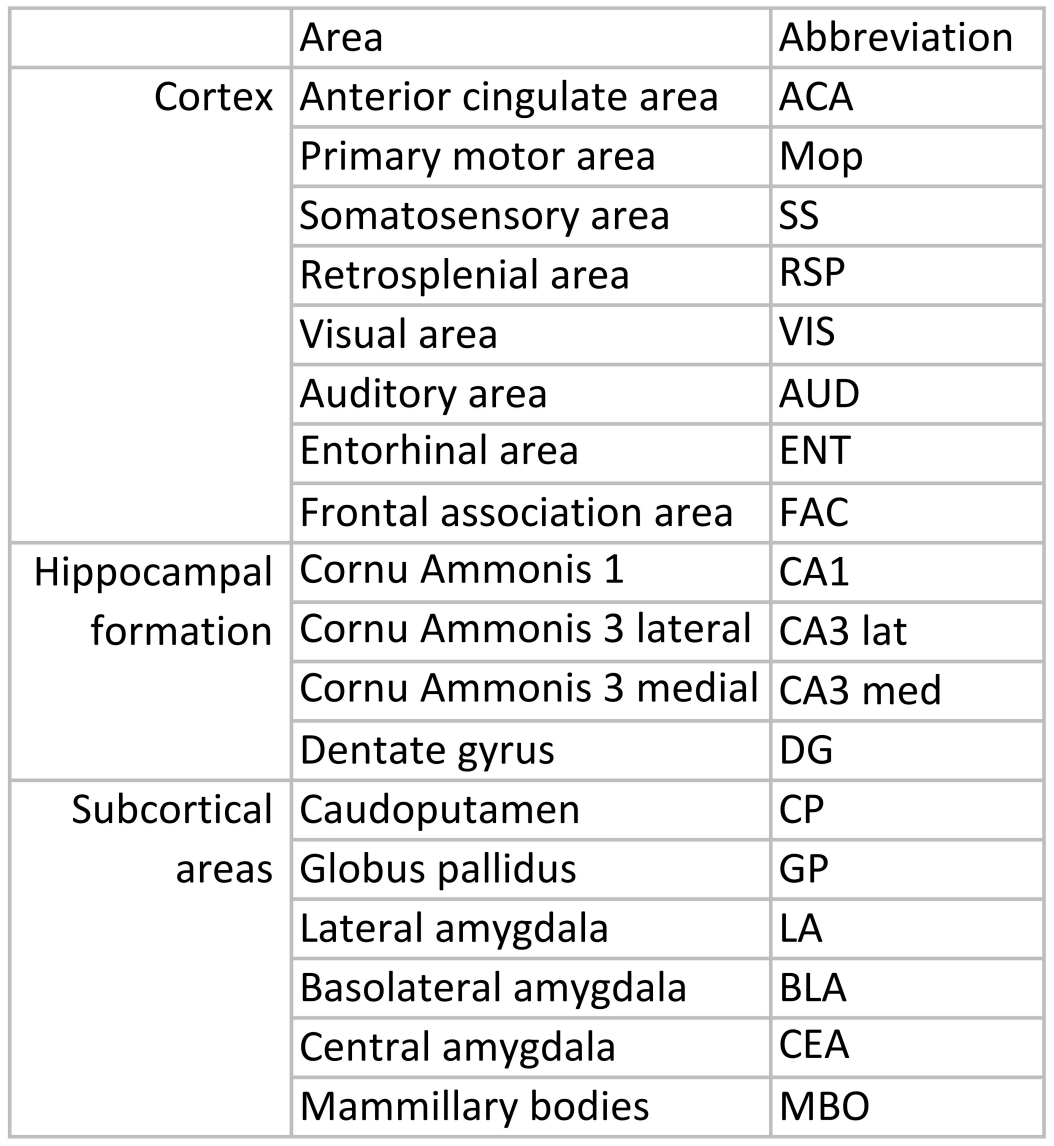
List over regions and abbreviations.

**Figure 4:**
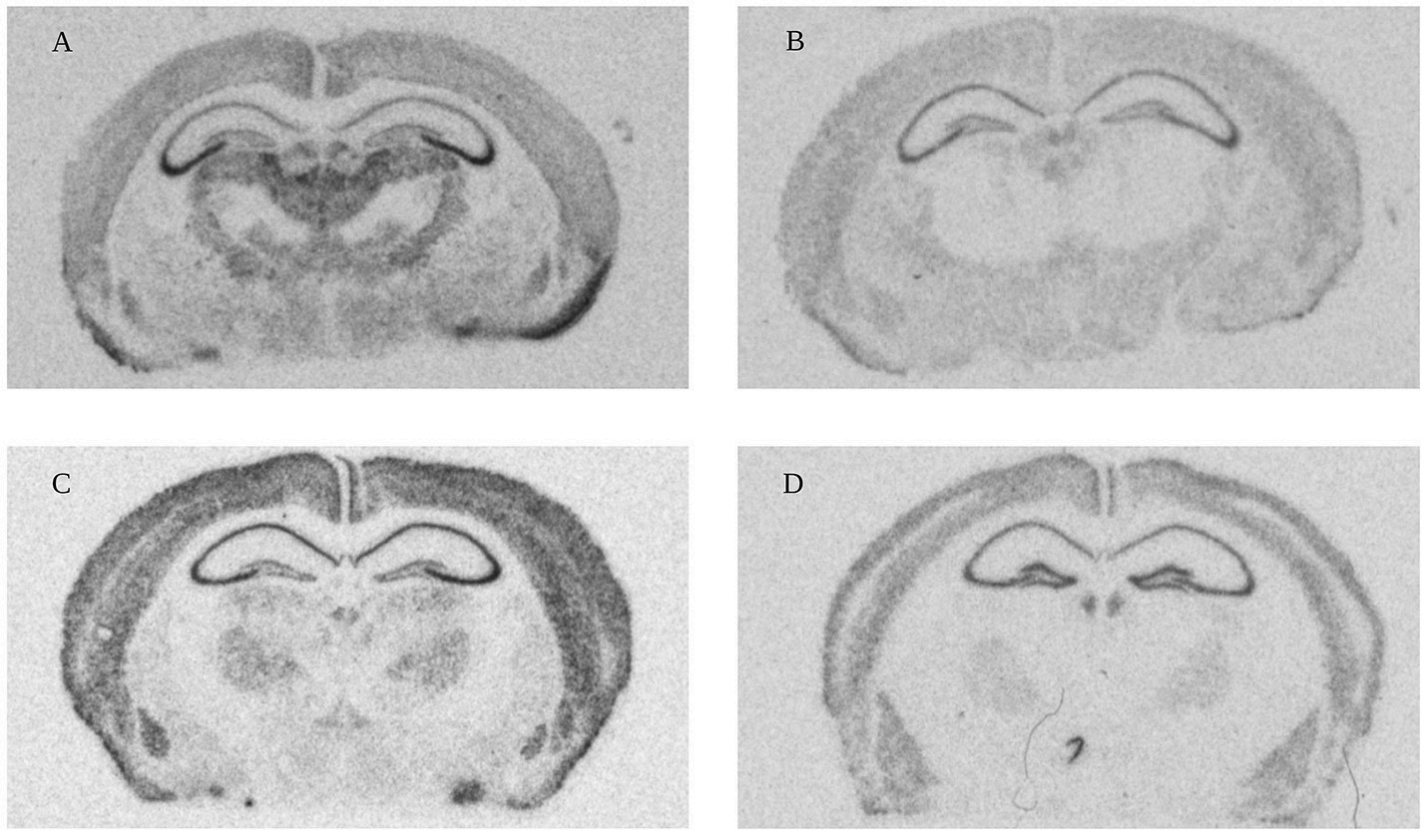
High resolution scans from X-ray film autoradiographs. Representative autoradiograms from *in situ* hybridization performed with ^33^P-labeled oligonucleotides for four different genes (**A**: Olfactomedin 1 mRNA 1 week; **B**: Nogo-A mRNA 2 weeks; **C**: Lingo-1 mRNA 4 weeks and **D**: NgR1 mRNA 104 weeks). These scans were used for measurements of gene expression in the 18 different regions (Figure 3).

Eighteen relevant brain areas (Figure 3), were chosen for analysis based on their function and identified using the appropriate neuroanatomical atlases (Paxinos and Franklin, 2007)^2^ Optical densities indicating presence of mRNA encoded by a gene of interest in manually defined areas were quantified using appropriate software (ImageJ, NIH). Example images used for measurements in Figure 4. A ^14^C step standard (Amersham, Biosciences Europe GmbH, Uppsala, Sweden) was included for each film to calibrate and to convert measured values into nCi/g. Expression levels were optained by converting the raw signal using the standard curve and adjusting for the level of isotope labeling, radioactive decay before and during film exposure, and for background detection levels. For each age and time point, 2–15 sections, average 5, were measured per region per animal.

### Statistics

Data were analyzed with a mixed model analysis and split per gene and group of regions (cortex, hippocampal formation and subcortical areas). If a significant effect was found, a *post-hoc* analysis was performed using the estimated marginal means. The following test were performed per gene and group: Each area was tested compared to all other areas in the region and for each timepoint. Furthermore, age groups were compared to their neighboring groups during postnatal development and maturation (P0 to 4 weeks) and all age groups were compared to the 14 week (adult) group. The *p*-values were then adjusted with Bonferroni correction for the number of tests per group. Statistical calculations were made in SPSS (SPSS 23, IBM, USA). Complete data are availible from the authors.

## Results

With quantitative *in situ* hybridization (ISH) we mapped the unique mRNA expression of 11 genes involved in Nogo-like signaling. In total around 21,000 manual measurements were peformed to cover 18 different brain areas at 6 points in time; P0, 1, 2, 4, 14, and 104 weeks. The regions were chosen to include areas responsible for motor-, sensory-, cognitive-, and memory functions; some regions are involved in more than one category: Motor functions: Primary motor area, globus pallidus and caudoputamen. Sensory functions: Somatosensory area, auditory area, visual area. Cognitive functions: Retrosplenial cortex, entorhinal cortex, basolateral-, lateral- and central amygdala, anterior cingulate area, frontal association area and mammillary bodies. Memory functions: CA1, medial CA3, lateral CA3, dentate gyrus (DG), mammillary bodies, entorhinal cortex, amygdala, frontal association cortex.

**Figure 5:**
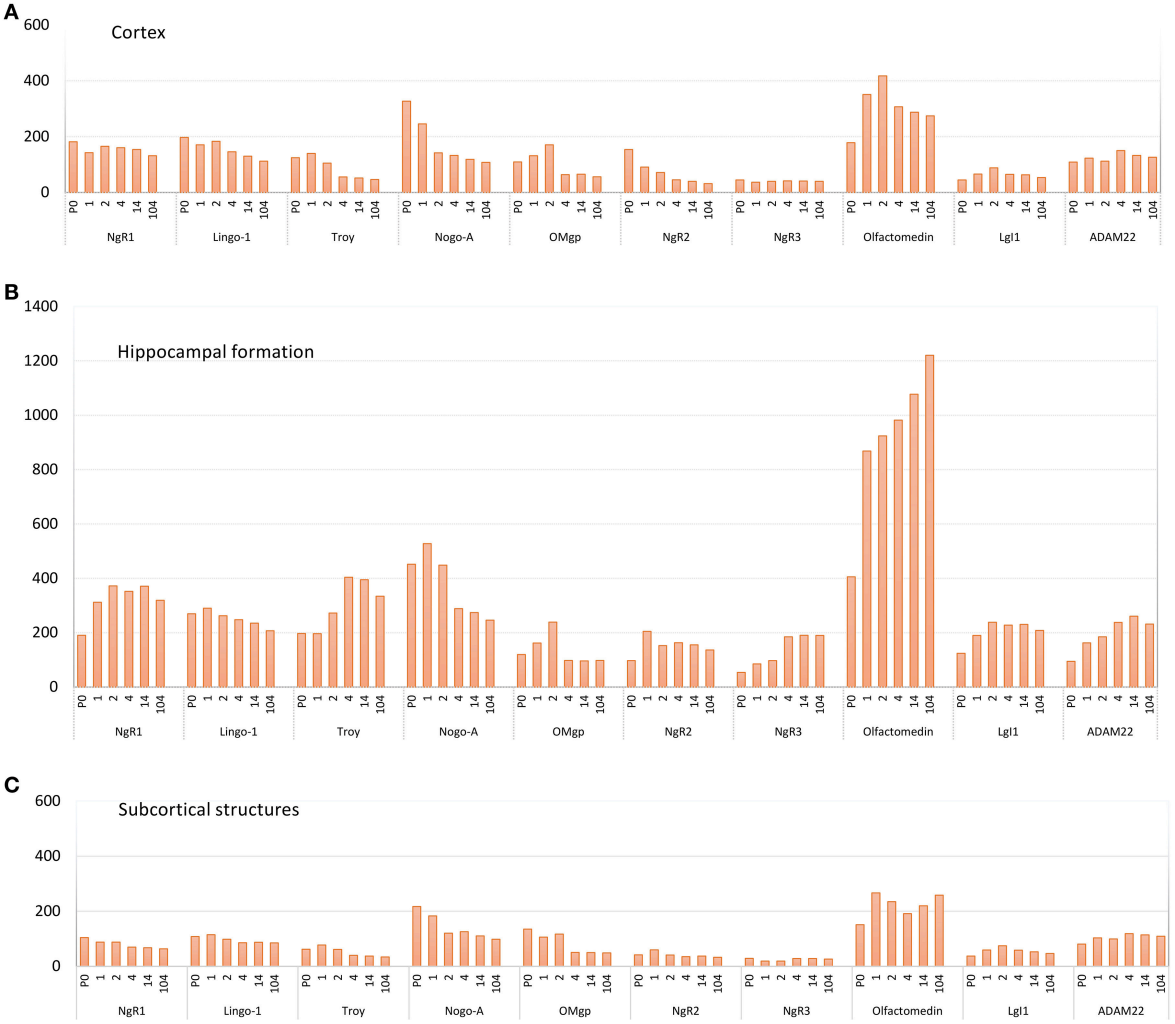
Pooled average expression of all subregions in the three main regions over time for each gene; **(A)** cortex, **(B)** hippocampal formation and **(C)** subcortical structures. Subregions are found in Figure 3. Y-axis: expression in nCi/g.

In each region we compared all ages to the adult (14 week) mice. We also compared values between each neighboring timepoint (P0 vs. 1 week, 1 week vs. 2 weeks, 2 weeks vs. 4 weeks, as well as regions against each other at each time-point. Changes and differences are mentioned if multiple comparison corrected *p*-values are <0.05. Due to the large number of significances (513), exact *p*-values are not presented below but are summarized in Figure 5.

### Expression over time and levels of expression in different regions compared to each another

**Figure 6:**
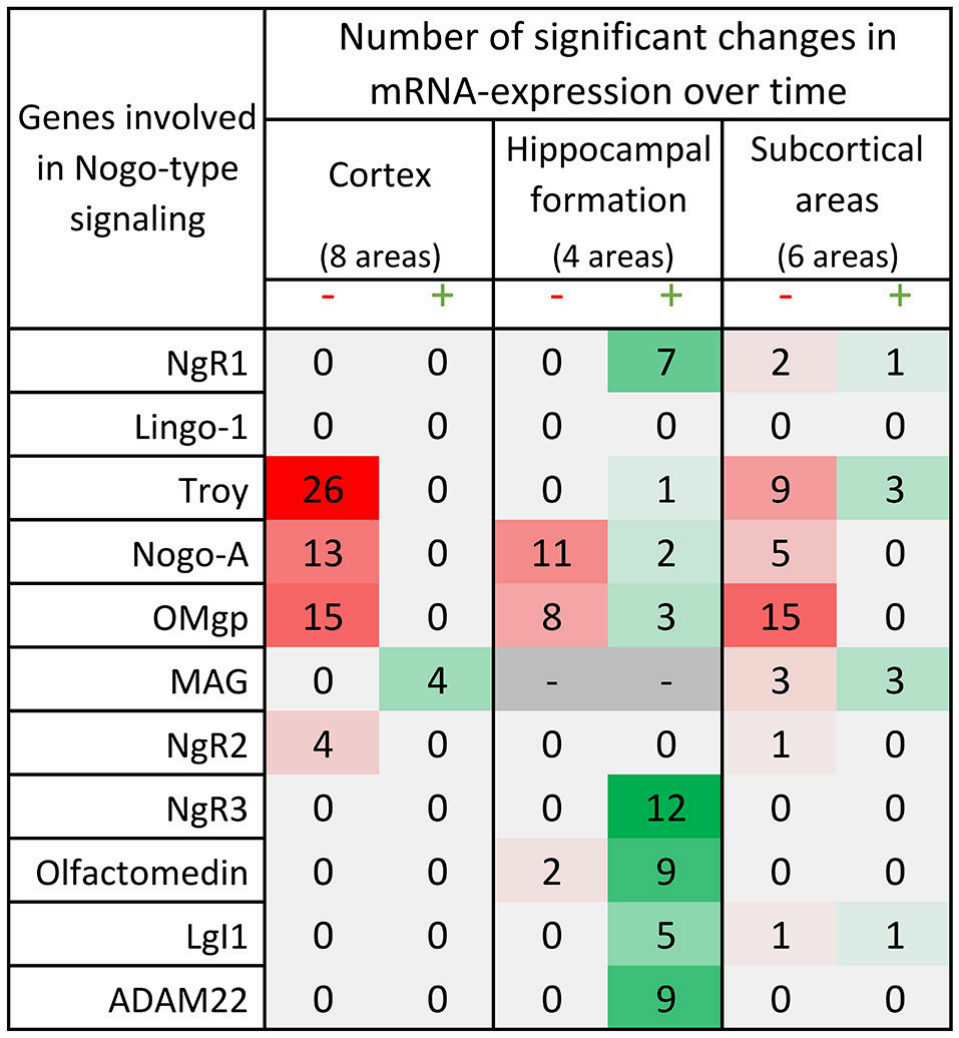
Distribution of 175 significant changes (*p* < 0.05) of mRNA expression over time for the 11 studied Nogo-related genes in the three main regions; cortex, hippocampal formation and subcortical areas. The changes are later described and visualized in charts in the paragraphs for each gene. In this table, significant change of mRNA expression between either neighboring time points (1 week – P0, 2 weeks – 1 week or 4 weeks – 2 weeks) or when comparing the mRNA expression in the adult 14 weeks old mice to that in P0, 1 or 2 weeks old mice, is coded using a green scale concerning increases, and decreases using a red scale. Notably, all genes except MAG decrease with time or lack a significant change in cortical areas, while hippocampal levels mostly increase with time. It is also the hippocampal formation that had significant changes in all but 2 of the investigated genes. Changes were detected with the help of a mixed model analysis, see statistics section.

**Figure 7:**
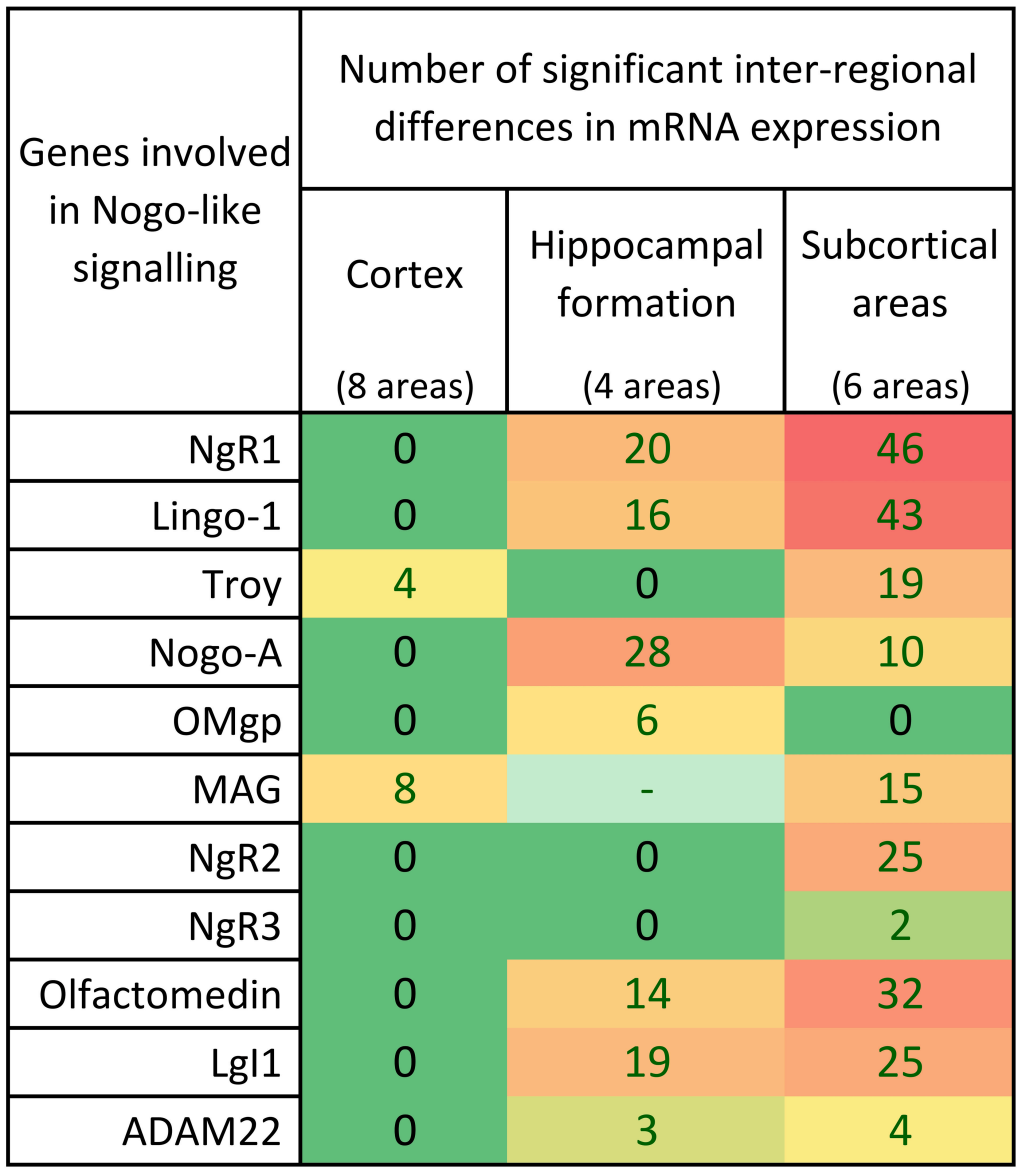
Distribution of 339 significant differences (*p* < 0.05) between regions for the 11 studied Nogo-related genes in cortex, hippocampal formation and subcortical areas. In the cortex, most of the genes had similar expression in most cortical areas investigated. In the hippocampus, 7/10 genes were differently expressed in the different regions while 3 had an even expression. Inter-regional mRNA expression level differences were foremost found in the heteregenous subcortical regions.

**Figure 8:**
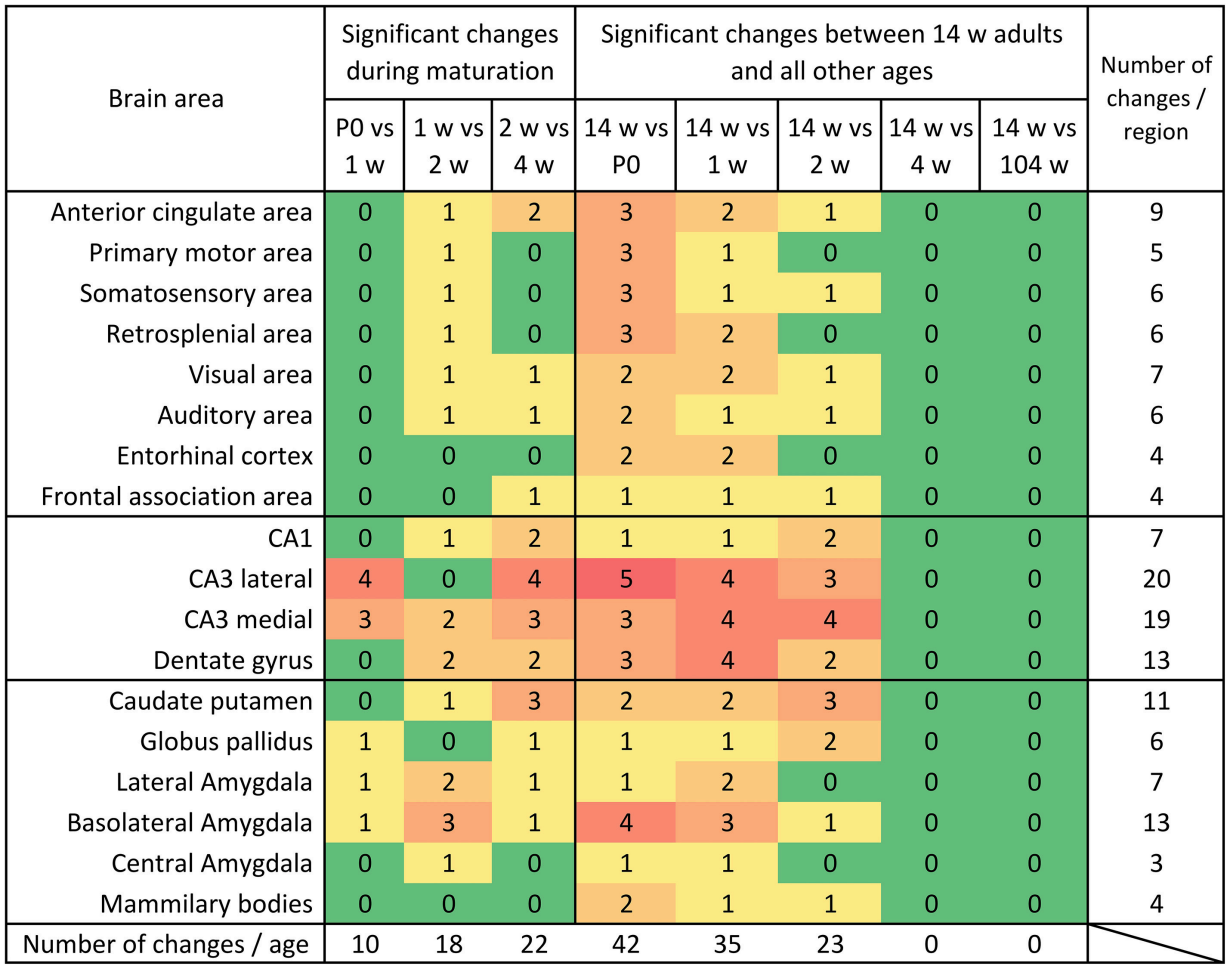
Distribution of significant changes (*p* < 0.05) of mRNA levels per region during maturation and aging. During maturation, most changes were observed between week 2 and 4. When comparing the mRNA expression levels in 14 weeks old mice to the young ages it becomes clear that the system gradually reaches its adult expression level between 2 and 4 weeks and remains remarkably stable into aging.

The 11 genes had various levels of mRNA expression. For instance, Olfactomedin had the highest levels of mRNA expression of the tested genes, while NgR3 mRNA levels were very low (Figure 5). Significant change of mRNA expression between time points are found in Figure 6. Inter regional differences are visualized in Figure 7, and areal distribution of mRNA changes in Figure 8.

### NgR1 and its coreceptors Lingo-1 and Troy

#### NgR1

**Figure 9:**
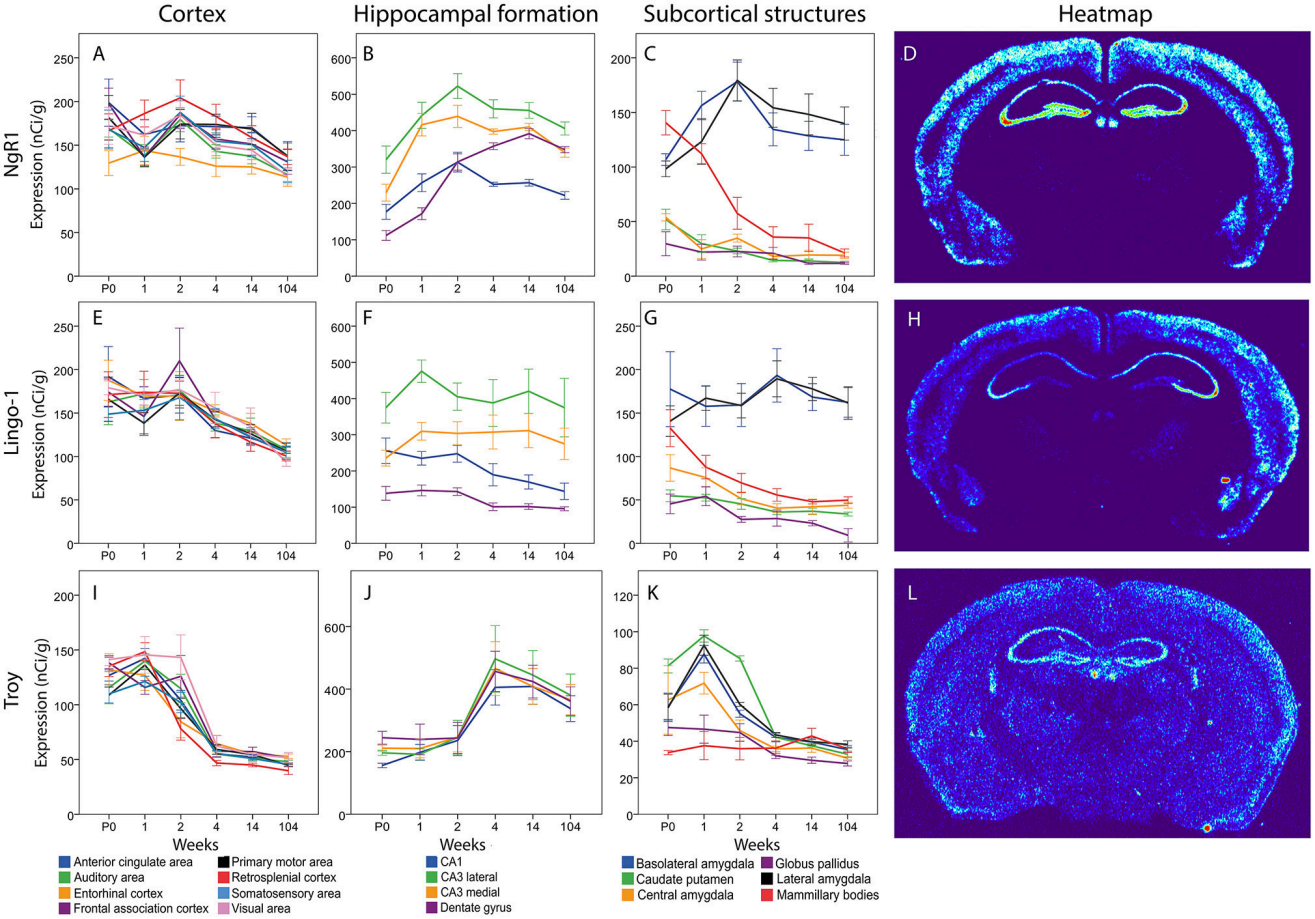
NgR1 **(A–C)**, Lingo-1 **(E–G)** and Troy **(I–K)** mRNA levels in 18 brain regions over time. The x-axis indicates age in weeks, the y-axis indicates levels of mRNA expression in nCi/g, and is individual for each gene and region. Significant changes defined as p < 0,05 are mentioned in the text for each gene. Heatmaps of the three genes **(D,H,L)** visualizing expression in 14 week old mice.

For the key receptor NgR1 we found an even mRNA expression in cortical areas with no significant change over time (see Figure 9). In hippocampus NgR1 increased during the first weeks of life in most studied areas. We found an increase from P0 to 1 week in lateral and medial CA3, and from 1 to 2 weeks in the DG). In medial and lateral CA3 areas and the DG we observed an increase from P0 to adulthood. However, CA1 expression did not change significantly during development and its expression was generally low in relation to the other structures. From birth until 2 weeks, lateral CA3 had a higher expression of NgR1 mRNA than DG, and throughout life CA3 expression was higher than that in CA1. Medial CA3 had a higher NgR1 mRNA expression than DG during the first 2 weeks of life, and its expression was higher than CA1 from 1 week onwards. DG had a higher mRNA expression than CA1 from 4 week throughout life. Subcortical areas had varied expression levels. Subcortical areas were mainly stable throughout life except for the LA which increased from 1 to 2 weeks and the mammillary bodies (MBO), in which NgR1 mRNA had decreased at 14 weeks as compared to its peak at P0 and 1 week. At P0 we found MBO having a stronger expression than GP, CEA and CP, while BLA and LA had higher expression than GP. From 2 weeks, BLA and LA had a strikingly higher expression than the other regions.

#### Lingo-1

Messenger RNA encoding Lingo-1 did not change significantly over time or between regions in the cortical areas. In hippocampus the Lingo-1 mRNA was generally steady over time. Lateral CA3 had a higher expression than DG at P0. At 1 week, the Lingo-1 mRNA level in lateral CA3 was higher than in all the other regions. From 2 weeks throughout life Lingo-1 mRNA in lateral CA3 was higher than in DG and CA1. Medial CA3 was higher than DG from 1 week until 14 weeks. BLA and LA had a strikingly higher expression than CP and GP throughout life. In subcortical areas BLA and LA had a strikingly stronger expression than other regions. BLA was stronger than CEA at all ages and from 2 weeks onwards. BLA was also stronger than MBO. From 1 week until 14 weeks LA was higher than CEA and MBO.

#### Troy

The Troy mRNA expression pattern differed markedly from that of NgR1 and Lingo-1. In cortical areas most regions, except EC Mpo and SSA, underwent a steep decrease of Troy mRNA from 1 to 4 weeks of age. All regions had a lower mRNA expression in adult than in young mice. In the hippocampal formation we saw a pattern where an increase occurs between weeks 2 and 4 albeit the only statistically significant change was in lateral CA3. In the subcortical regions we found a peak in several areas at 1 week and all areas had a very low expression in the adult mouse. BLA and LA Troy mRNA levels increased from P0 to 1 week and later, together with CEA, decreased from 1 to 2 weeks, while CP and Troy mRNA levels decreased from 2 to 4 weeks. BLA, LA, CEA and CP Troy mRNA levels were lower in the adult than during postnatal development. CP had a higher expression than all other areas except CEA at P0. At 1 week BLA was stronger than GP and MBO while CP and LA were stronger than CEA, GP and MBO. CEA was also stronger than GP and MBO at 1 week. At 2 weeks CP was stronger than all the other areas.

### Neurite-growth inhibitory Ligands Nogo-A, OMgp and MAG

#### Nogo-A

**Figure 10:**
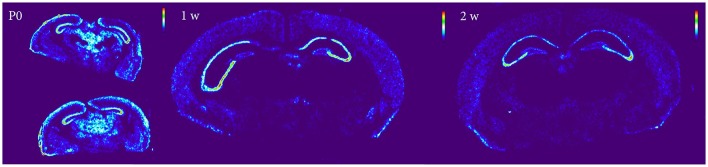
Heatmap of Nogo-A sections at P0, 1 and 2 weeks, demonstrating a global decline of mRNA expression during postnatal development.

In cortical areas Nogo-A mRNA was strongly expressed at P0 and levels in all areas except FAC decreased significantly over time (Figure 10). In the hippocampal formation, Nogo-A mRNA levels increased in medial and lateral CA3 from P0 to 1 week, to later, together with CA1, decrease from 2 to 4 weeks of age. CA1 also decreased from 2 to 4 weeks and CA1 and both CA3 regions had a lower expression at 14 weeks than during the first weeks of life. Lateral CA3 had a generally higher mRNA expression than the other regions. It was stronger than CA1 throughout life and stronger than medial CA3 from P0 to 2 weeks. Medial and lateral CA3 were significantly higher than DG throughout life. Medial CA3 had a stronger expression than CA1 from 1 week onwards. CA1 was higher than DG at P0 and 1 week. In subcortical areas Nogo-A mRNA levels decreased with age in BLA, LA and MBO and BLA also decreased from 1 to 2 weeks. At P0, BLA, and MBO had a higher expression than CP. At 1 week BLA, LA and MBO had a higher expression than CP, CEA, and GP.

#### OMgp

**Figure 11:**
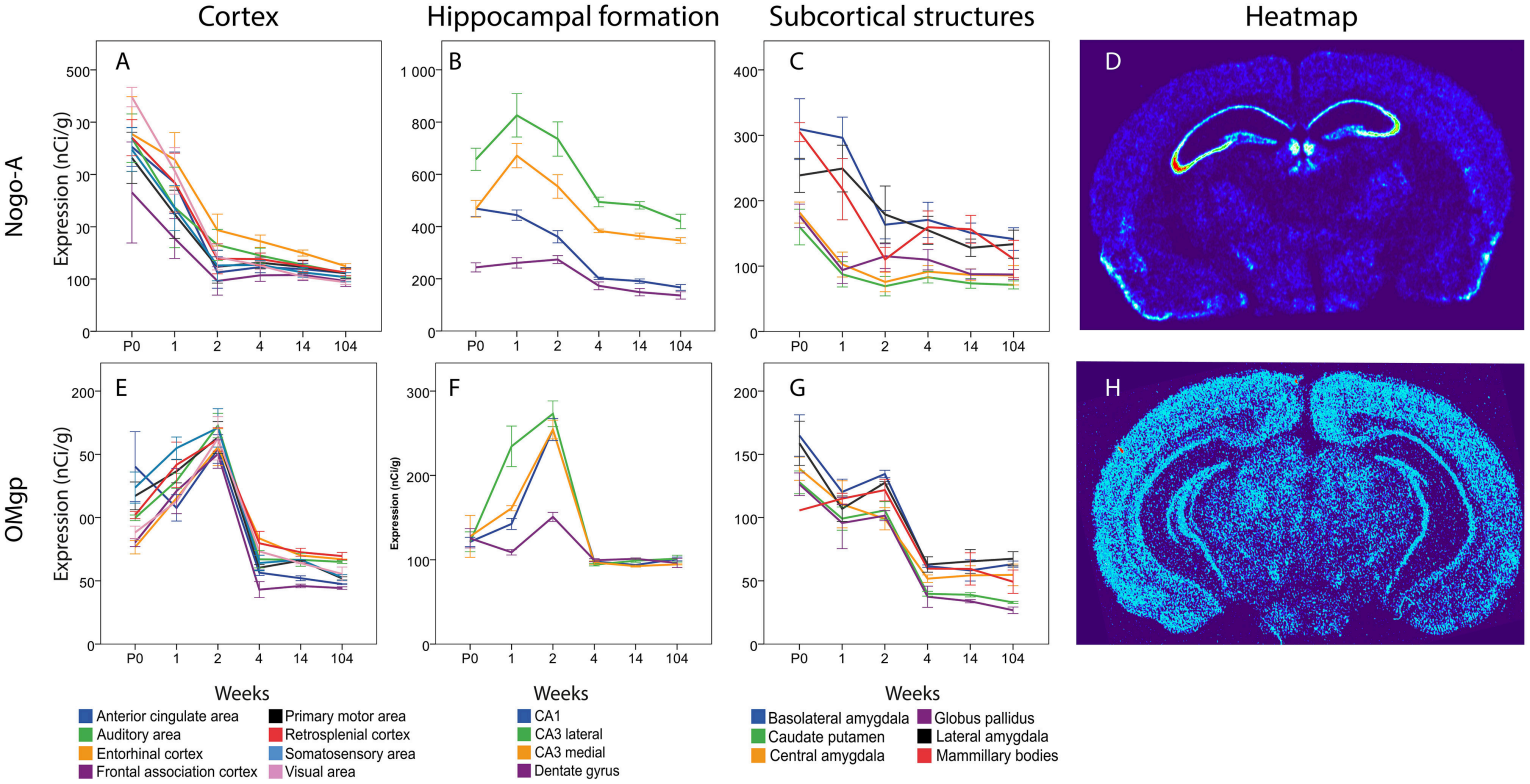
Graphs showing expression levels of mRNA encoding Nogo-A **(A–C)** and OMgp **(E–G)** in 18 cerebral regions over time. For full legend see Figure 9. Heatmaps of the two genes **(D,H)** visualizing expression in 14 week old mice.

In cortical areas OMgp mRNA was found at increasing levels until 2 weeks of age, and later dropped profoundly as the mice matured into adults (Figure 11). All cortical regions except VIS and EC decreased significantly from 2 to 4 weeks. All regions had a lower expression in the adult than in the 2 weeks old mouse. In the hippocampal region we noted the same pattern with an initial increase during the first 2 weeks until the expression of OMgp mRNA dropped to remain on low and steady levels throughout life. We also found lateral CA3 to increase from P0 to 1 week while CA1 and medial CA3 had a later increase from 1 to 2 weeks. These regions then decreased from 2 to 4 weeks. At 1 week lateral CA3 had a higher expression than the other areas. At 2 weeks all regions were higher than DG. In subcortical regions two stages of decreased expression were identified, first from P0 to 1 week and then from 2 to 4 weeks. Significant changes were identified in LA which decreased from P0 to 1 week and BLA, CP, GP, LA, GP, which decreased from 2 to 4 weeks. All genes had a lower expression in adulthood than in early life.

#### MAG

(Data not presented) As the expression of MAG naturally follows the myelination of the CNS (Inuzuka et al., 1991), no expression was found before 1 week and MAG was non-detectable in hippocampus. In cortical areas MAG mRNA levels were generally low throughout life and we could not detect any expression in FAC at 1 week. MAG mRNA in SS, MPo and AA increased from 1 to 2 weeks and ACC increased from 2 to 4 weeks. CP and GP increased strongly in subcortical areas from 1 to 2 weeks to directly decrease from 2 to 4 weeks. GP had a higher expression at 14 weeks than at 1 week when very low MAG mRNA levels were detected.

### Receptors NgR2 and NgR3

#### NgR2

**Figure 12:**
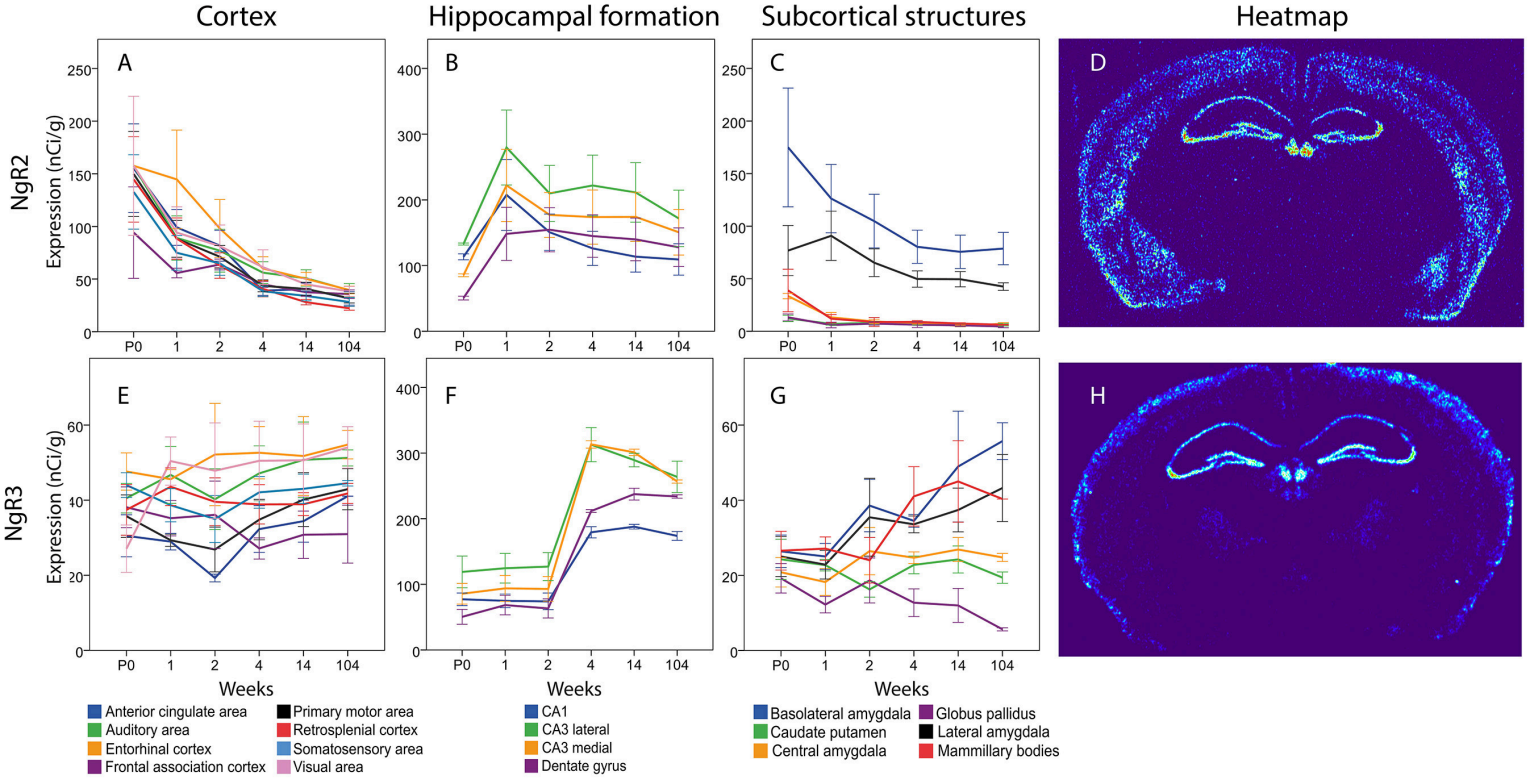
Graphs showing the mRNA expression for NgR2 **(A–C)** and NgR3 **(E–G)** in the 18 cerebral regions over time. For full legend see Figure 9. Heatmaps of the two genes **(D,H)** visualizing expression in 14 week old mice.

The total expression of NgR2 mRNA tended to decline over time (Figure 12). In cortical areas, ACA, Mop, SS and RSP mRNA levels were lower at 14 weeks than at P0. In the hippocampal formation, the overall impression was that NgR2 mRNA levels tended to increase during the first week of life, and thereafter level off or decrease modestly. However, none of these changes were significant. In subcortical areas, levels of NgR2 mRNA were robust in BLA and LA and low or non-detectable in other areas. Statistical analysis also showed that BLA NgR2 mRNA levels were significantly higher than those in other areas at P0. At 1 and 2 weeks it was stronger than all other areas except LA. Throughout life BLA had a stronger expression of NgR2 mRNA than CP and CEA. LA NgR2 mRNA levels in turn, were significantly higher than those in CP, GP, CEA, and MBO at 1 week. BLA NgR2 mRNA levels were lower in adult than in young mice.

#### NgR3

Generally, NgR3 mRNA was present in low levels in the developing and adult brain, although with a tendency to increase between the second and fourth week of life in the hippocampal formation and in some subcortical areas (Figure 12). Statistical analysis revealed that medial and lateral CA3 and DG increased from 2 to 4 weeks, leading to a stronger NgR3 mRNA expression in adulthood than at P0, 1 and 2 weeks in these three areas. No change in mRNA expression was detected over time for NgR3 in subcortical areas. At 14 and 104 weeks BLA had a stronger NgR3 mRNA expression than GP.

### Nogo-signaling inhibitors: olfactomedin, LgI1 and ADAM22

#### Olfactomedin

**Figure 13:**
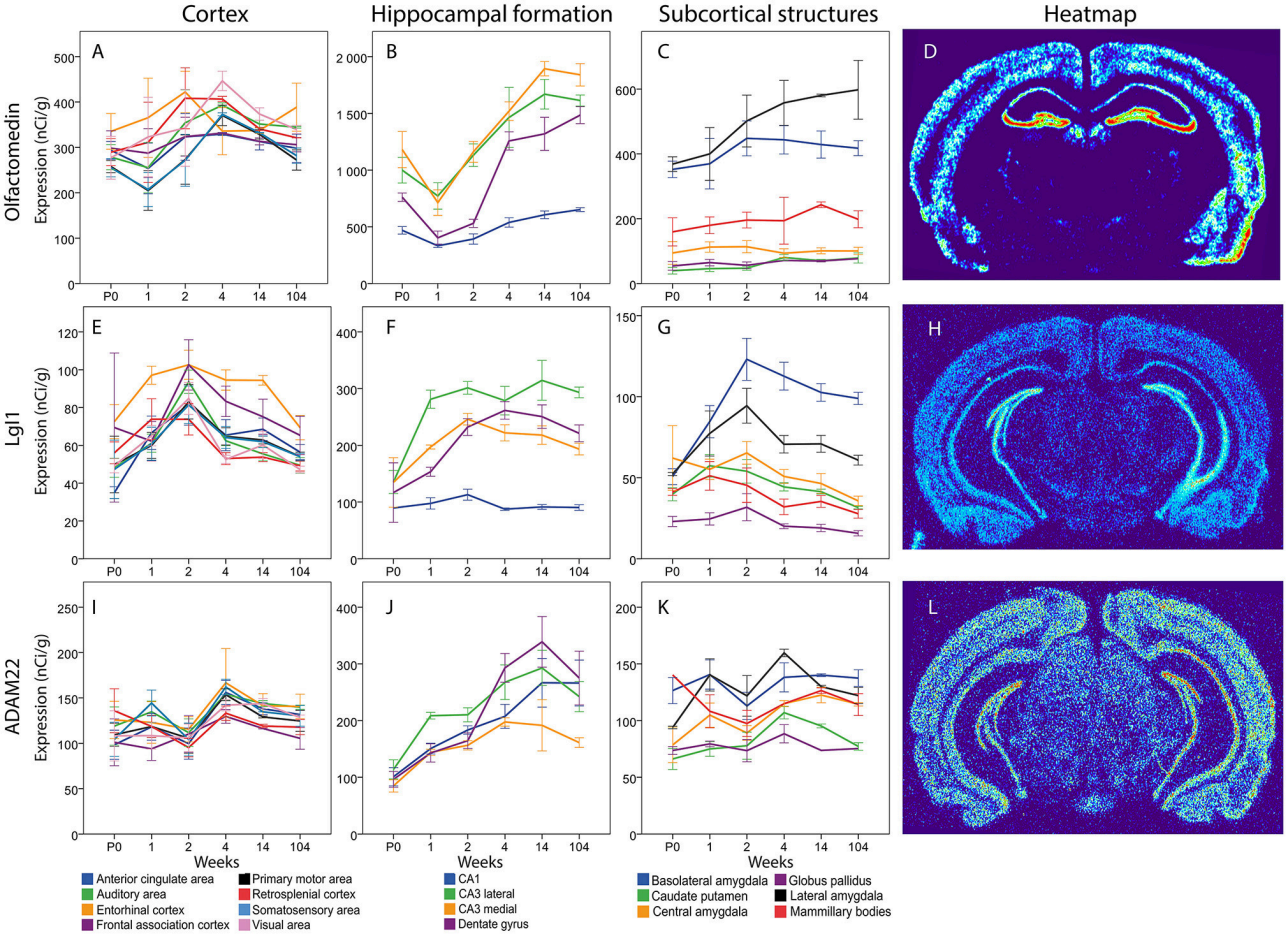
Graphs showing the mRNA expression for Olfactomedin **(A–C)**, LgI1 **(E–G)** and ADAM22 **(I–K)** in 18 cerebral regions over time. For full legend see Figure 9. Heatmaps of the three genes **(D,H,L)** visualizing expression in 14 week old mice.

Messenger RNA encoding olfactomedin was robustly present in all cortical areas from birth to old age (Figure 13). There were no significant changes across time and no marked differences between cortical areas. In the hippocampal formation there was a general tendency for Olfactomedin mRNA levels to increase from the first postnatal weeks to adulthood. Medial CA3 decreased from P0 to 1 week to later again increase from 1 to 2 weeks. DG olfactomedin mRNA increased from 2 to 4 weeks. DG, Lateral and medial CA3 had a higher expression at 14 weeks compared to the young mouse. Lateral and medial CA3 had a stronger expression than the stable CA1 throughout life. At 2 weeks, both lateral and medial CA3 also had a stronger expression than DG. At 104 weeks all regions had a stronger expression than CA1. Among subcortical areas, lateral and basolateral amygdala stood out as expressing higher levels of olfactomedin mRNA than other analyzed areas. BLA had a stronger expression than CP, CEA and GP from P0 to 2 weeks. At 2 weeks it was also stronger than MBO. LA had a stronger expression of Olfactomedin mRNA than CEA, CP, GP and MBO throughout life (no significance at 4 and 14 weeks for MBO).

#### LgI1

Overall, there was a tendency for cortical expression of LGI1 mRNA to increase from low levels at birth to 3–4-fold higher levels at 2 weeks of age, after which levels declined the next 2 weeks with modest changes thereafter (Figure 13). In hippocampus lateral CA3 LgI1 mRNA levels increased from P0 to 1 week while the expression in DG increased from 1 to 2 weeks. Both stayed at higher levels throughout life. At 1 week lateral CA3 had a higher expression of LgI1 mRNA than the other regions and medial CA3 was higher than CA1. From 2 weeks and throughout life all other regions of the hippocampal formation had higher LgI1 mRNA expression levels than CA1. At 14 and 104 weeks lateral CA3 had a higher expression than medial CA3. In subcortical regions, LgI1 mRNA levels increased in BLA from 1 to 2 weeks. Notably, BLA was the only region that had a higher expression in adulthood than at early ages. At 1 week, BLA, and LA had a stronger expression of LgI1 mRNA than GP. From 2 weeks and onwards BLA had a stronger expression than all the other regions except LA. At 2 week LA had a stronger LgI1 mRNA expression than CP, GP and MBO. At 4, 14, and 104 weeks LA had a stronger expression than GP.

#### ADAM22

The levels of Adam22 mRNA were stable in all cortical areas from birth to old age. In contrast, Adam22 mRNA levels in all regions of the hippocampal formation increased with age, with a tendency to decrease at old age. At 1 week, medial and lateral CA3 were found to have a higher expression than CP and GP (Figure 13).

## Discussion

The complex Nogo-like growth inhibitory signaling occurs between neurons as well as between neurons and glial cells and the extracellular matrix (Josephson et al., 2001; Domeniconi et al., 2002; Mi et al., 2004; Park et al., 2005; Venkatesh et al., 2005; Zhang et al., 2009; Cafferty et al., 2010; Dickendesher et al., 2012; Nakaya et al., 2012; Ahmed et al., 2013; Kurihara et al., 2014; Lovero et al., 2015). The many gene products involved in Nogo-like signaling offer rich opportunities to target signaling, and, in particular, to inhibit signaling in order to improve axon regeneration and structural synaptic plasticity. This could be beneficial for human pathologies such as spinal cord injury, stroke, traumatic brain injury, multiple sclerosis, dementia, retinopathies, and developmental disturbances (Kim et al., 2004; Satoh et al., 2005; Fontoura and Steinman, 2006; Zhou et al., 2011; Lee and Petratos, 2013; Lindau et al., 2014; Liu et al., 2015; Sozmen et al., 2016; Pernet, 2017; Rodriguez et al., 2017). To help design such treatments, we need to know where the Nogo-like signaling genes are expressed, and how they are regulated together.

The scope of the present study was to investigate the transcriptional age related regulation of genes involved in Nogo-like signaling, as reflected by amounts of mRNA, to inform about the activity of the specific genes of interest. The use of stringent *in situ* hybridization, and well characterized radioactive oligoprobes, allows for quantitative comparisons across our material. While alterations of mRNA levels often correlate to altered levels of the corresponding proteins, we are well aware that this is not always the case. However, similar quantitative comparisons across proteins using immunohistochemistry is currently not possible, due to variations in antibody specificities and epitope availabilities, as well as the fact that antibodies suited for immunohistochemistry are not available for all studied genes.

Nogo-A and OMgp proteins are present in both neurons and myelin, MAG protein in myelin only. Thus, the corresponding Nogo-A, OMgp, and MAG mRNA species are expected to be located in the oligodendroglial cytoplasm. However, these parts of the oligodendroglial cells constitute a very small percentage of areas of gray matter tissue studied. It is thus unlikely that oligodendroglial Nogo-A, OMgp, or MAG mRNA would contribute significantly to the densitometry measurements in gray matter. This view is in line with the fact that mRNA encoding MAG, present only in myelin/oligodendroglial cells, was very difficult to detect, or sometimes undetectable in our study.

We recently described the location of expression and transcriptional response to strong neuroexcitation of 11 key Nogo-like signaling genes in nine brain areas of adult mice (Karlsson et al., 2017). Here we used quantitative *in situ* hybridization to monitor 11 Nogo-like signaling genes from birth to old age in eight cortical, four hippocampal and six subcortical mouse brain areas.

Overall, we found profound differences between brain regions in hippocampal and subcortical areas. The levels of expression were highly dynamic during the first weeks of life to later stabilize at adult levels by 4 weeks of age. The amount of mRNA differed between analyzed genes, ranging from Olfactomedin 1 mRNA which was present in the highest amounts (up to 1,200 nCi/g), to NgR3 mRNA which was always expressed at low levels (max < 200 nCi/g).

In the eight studied cortical areas there was a decrease of levels of mRNA encoding Nogo-A, NgR2, Lingo-1, and Troy from birth to adulthood, while levels of NgR1, olfactomedin 1, NgR3, and ADAM22 were mostly stable from birth into old age. In all eight cortical areas, Nogo-A mRNA levels were high at birth and decreased during the first 2 weeks of life, after which steady levels were maintaind until old age. Differences between adulthood (14 weeks) and old age (104 weeks) were lacking. We also noted that mRNA levels at 2 weeks of age often tended to differ from levels at 1 and 4 weeks, suggesting a period around 2 weeks to be particularly dynamic in terms of an influence of Nogo-like signaling on development. Taken together, the cortical presence of mRNA encoding all 11 genes suggests key roles of Nogo-like signaling for structural synaptic plasticity in cortical areas during development, and to regulate plasticity in the adult and aging brain as needed to form lasting memories. It is tempting to speculate that during development, when the brain undergoes strong growth and is supported by high amounts of growth factors, the growth processes are kept under control through a correspondingly strong Nogo-like signaling. This is compatible with the finding that knockdown of LOTUS results in a widening of the lateral olfactory tract (Sato et al., 2011).

In contrast to the cortical areas, mRNA levels in hippocampal areas increase from birth to 1 week for several genes (NgR1, Nogo-A, OMgp, LgI1, ADAM22, NgR2). Overall, lateral CA3 express higher levels of mRNA encoding the investigated genes, followed by medial CA3. However, almost every investigated gene gave rise to a gene-specific pattern of mRNA levels for the four measured areas (CA1, medial and lateral CA3, DG) and across the six ages. OMgp mRNA differed the most, by having a peak at 2 weeks in the hippocampus proper, that decreased sharply to similar levels as the DG by 4 weeks that remained until 104 w. Generally, the hippocampal formation expressed higher levels of Nogo-like signaling genes than cortical areas, suggesting key roles of Nogo-like signaling in the hippocampal formation and increasingly so in adult, compared to developing mice.

It is notable that olfactomedin mRNA, which is the most abundant mRNA species we analyzed, is reported to be lacking in CA2 and Purkinje neurons (Karlsson et al., 2017)^1^. We speculate that the lack of olfactomedin in CA2 neurons, which are also embedded in an unusually robust perineuronal net, and in Purkinje neurons, contributes to rendering these neurons less prone to undergo structural alterations in response to increased activity. In the investigated subcortical areas, there was more variation between genes and in-between areas than in cortical and hippocampal areas, presumably because the chosen subcortical areas were anatomically/functionally more different than the chosen cortical and hippocampal areas. Interestingly, levels of mRNA encoding five of the investigated genes (NgR1, Lingo-1, olfactomedin, LgI1, NgR2) were higher in basolateral and lateral amygdala, and relatively high levels were also noted in these two areas for four additional genes (Nogo-A, ADAM22, Troy, OMgp). This suggests an important role for Nogo-like signaling for regulation of structural synaptic plasticity in basolateral and lateral amygdala, two structures important for memory and strong emotions (Goosens and Maren, 2001; Gale et al., 2004; Erlich et al., 2012; Kim et al., 2016).

Our study included direct comparisons between 3 months old and 2 years old mice analyzed together. The latter age is considered as an old age in mice, corresponding to geriatric conditions in humans. It was striking that there were no marked differences in levels of mRNA encoding any of the investigated genes between 3 months and 2 years old animals. This study has not investigated the span between 3 months and 2 years of age. Considering the fact that we found no significant differences between 4 weeks, 3 months and 2 years of age we find it unlikely that dramatic changes would occur in adulthood.

Our findings confirm and expand existing knowledge (Habib et al., 1998; Josephson et al., 2001, 2002; Vourc'h et al., 2003; Okafuji and Tanaka, 2005; Venkatesh et al., 2005; Barrette et al., 2007; Funahashi et al., 2008; Haybaeck et al., 2012; VanGuilder Starkey et al., 2013) regarding the expression of genes involved in Nogo-like signaling during postnatal development, in the adult and the aged mouse brain. Together, these findings reveal gene- area- and age-specific roles of these genes, and suggests stronger roles for Nogo-like signaling in areas in which structural synaptic plasticity is important.

## Conclusions

Expression of Nogo related genes undergo marked gene- and region-specific regulation during postnatal development. In a second stage, from 4 weeks onwards via 14 weeks and until 2 years of age mRNA levels of genes involved in Nogo-like signaling are mostly stable. In cortical areas, NgR1 and Lingo-1 were stably expressed, while the expression of Troy decreased to a low level maintained during adulthood and aging. Ligands Nogo-A and OMgp also decreased to a low level from week 4, while MAG increased strongly into adulthood. NgR2 decreased into adulthood, NgR3 was stable over life at a low level. In the hippocampal formation, NgR1 and Troy increased with age, while Lingo-1 was stable at different levels in different hippocampal areas. Both OMgp and Nogo-A decreased with age, while MAG levels were too low to detect. NgR2 was stable, while NgR3 increased into adulthood. Olfactomedin levels decreased after birth to later increase robustly until 14 weeks and remain at high levels. The LgI1/ADAM22 complex showed increased expression until adulthood. Subcortical areas had the largest inter-regional differences with higher levels of most investigated genes in lateral and basolateral amygdala.

The widespread differences and the unique expression patterns of the different genes involved in Nogo-like signaling strongly suggest that the functional complexes could look vastly different in different areas. This study suggests that the inhibitory mileu is much more area specific than what has previously been assumed.

## Author contributions

GS, TK, and LO designed the project. TK collected the material. GS analyzed the material and collected the data. GS and TK performed the statistical tests. GS, TK, and LO interpreted the data and wrote the manuscript. All authors gave final approval of the manuscript.

### Conflict of interest statement

The authors declare that the research was conducted in the absence of any commercial or financial relationships that could be construed as a potential conflict of interest.

## Footnotes

^1^Allen Brain atlas ISH Data. *Allen Brain Atlas: Mouse Brain* Available online at http://mouse.brain-map.org/.

^2^Allen Brain Atlas. Available online at: http://atlas.brain-map.org/atlas.
